# α-Tubulin Acetyltransferase Is a Novel Target Mediating Neurite Growth Inhibitory Effects of Chondroitin Sulfate Proteoglycans and Myelin-Associated Glycoprotein

**DOI:** 10.1523/ENEURO.0240-17.2018

**Published:** 2018-02-28

**Authors:** Victor S. C. Wong, Cristina Picci, Michelle Swift, Max Levinson, Dianna Willis, Brett Langley

**Affiliations:** 1 The Burke Medical Research Institute, White Plains, NY 10605; 2 Brain and Mind Research Institute, Weill Cornell Medicine, New York, NY 10065; 3 Health Sport and Human Performance, University of Waikato, Hamilton 3240, New Zealand

**Keywords:** α-tubulin acetylation, α-tubulin acetyltransferase, chondroitin sulfate proteoglycan, myelin-associated glycoprotein

## Abstract

Damage to the CNS results in neuronal and axonal degeneration, and subsequent neurological dysfunction. Endogenous repair in the CNS is impeded by inhibitory chemical and physical barriers, such as chondroitin sulfate proteoglycans (CSPGs) and myelin-associated glycoprotein (MAG), which prevent axon regeneration. Previously, it has been demonstrated that the inhibition of axonal histone deacetylase-6 (HDAC6) can promote microtubule α-tubulin acetylation and restore the growth of CSPGs- and MAG-inhibited axons. Since the acetylation of α-tubulin is regulated by two opposing enzymes, HDAC6 (deacetylation) and α-tubulin acetyltransferase-1 (αTAT1; acetylation), we have investigated the regulation of these enzymes downstream of a growth inhibitory signal. Our findings show that exposure of primary mouse cortical neurons to soluble CSPGs and MAG substrates cause an acute and RhoA-kinase-dependent reduction in α-tubulin acetylation and αTAT1 protein levels, without changes to either HDAC6 levels or HDAC6 activity. The CSPGs- and MAG-induced reduction in αTAT1 occurs primarily in the distal and middle regions of neurites and reconstitution of αTAT1, either by Rho-associated kinase (ROCK) inhibition or lentiviral-mediated αTAT1 overexpression, can restore neurite growth. Lastly, we demonstrate that CSPGs and MAG signaling decreases αTAT1 levels posttranscriptionally via a ROCK-dependent increase in αTAT1 protein turnover. Together, these findings define αTAT1 as a novel potential therapeutic target for ameliorating CNS injury characterized by growth inhibitory substrates that are prohibitive to axonal regeneration.

## Visual Abstract

**Figure:**
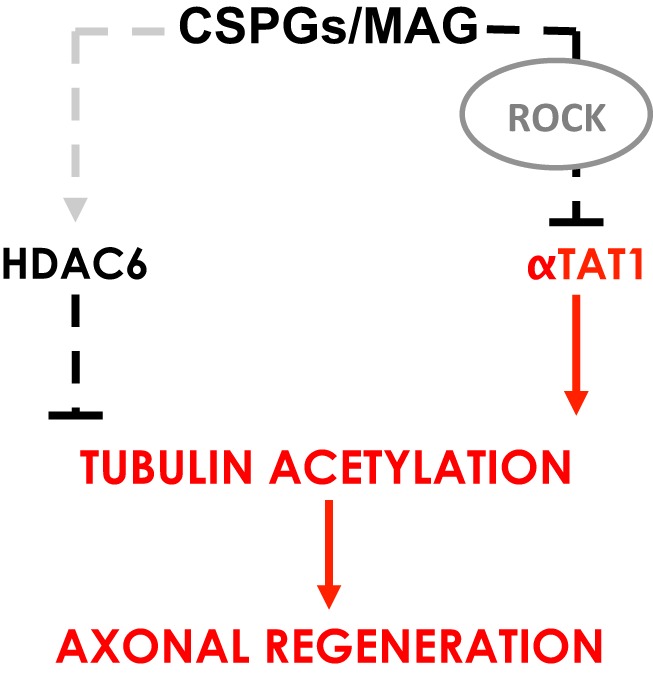

## Significance Statement

Chondroitin sulfate proteoglycans (CSPGs) and myelin-associated glycoprotein (MAG) represent significant barriers to axon regeneration after CNS injury. Inhibition of axonal histone deacetylase-6 (HDAC6), an enzyme that regulates α-tubulin deacetylation, has been shown to overcome the inhibitory effects of CSPGs and MAG to axon growth. In the present study, we report that αTAT1, the α-tubulin acetyltransferase that opposes HDAC6’s activity, is downregulated in neurites by CSPGs and MAG in cortical neurons *in vitro*. This reduction is associated with a loss of α-tubulin acetylation and occurs via a RhoA-kinase-dependent pathway. Restoring αTAT1 expression in CSPGs- or MAG-inhibited cortical neurons rescues neurite growth. Our results suggest that αTAT1 is a potential therapeutic target to promote axonal regeneration in the CNS.

## Introduction

CNS function requires the maintenance of axonal structural integrity and proper connectivity. As such, injury to axons often results in dysfunction, typified by the motor and sensory loss seen following spinal cord injuries. Exacerbating the consequences of injury, axonal regeneration in the CNS is limited, which results in the dysfunction becoming permanent (Dell'Anno and Strittmatter, 2017). Both intrinsic and extrinsic neuronal mechanisms contribute to failed axonal regeneration (Yiu and He, 2006). Many extrinsic factors are a result of the injury environment and are regarded to be prohibitive to axon regrowth. These include, but are not limited to, chondroitin sulfate proteoglycans (CSPGs; McKeon et al., 1999; Jones et al., 2003; Tang et al., 2003), and myelin associated-glycoprotein (MAG; McKerracher et al., 1994; Mukhopadhyay et al., 1994). These factors induce signaling, via RhoA and Rho-associated kinase (ROCK), which converges on the cytoskeletal network to inhibit axon growth (Dergham et al., 2002; Borisoff et al., 2003; Monnier et al., 2003; Mimura et al., 2006). Microtubules, which consist of cylindrical structures assembled from protofilaments of α- and β-tubulin heterodimers (Desai and Mitchison, 1997) and constitute a major component of the cellular and axonal cytoskeleton, play a critical role in axon extension and retraction. Microtubule lengths are variable, depending on the degree of assembly and disassembly at their plus and minus ends, making them highly dynamic. This dynamic structure is essential for many important cellular functions (Westermann and Weber, 2003), so it is not surprising that microtubules are under heavy and stringent regulation.

Posttranslational modification is a well-established mechanism of regulating microtubules dynamics, and this includes acetylation of α-tubulin on lysine residue 40 (K40; Nogales et al., 1998; Janke and Bulinski, 2011). The importance of α-tubulin K40 acetylation is underscored by several studies that reveal its role in promoting axonal transport, motor protein binding, and motility (Reed et al., 2006; Dompierre et al., 2007; Hammond et al., 2010; Alper et al., 2014; Godena et al., 2014). Using cell culture models, it has been shown that defective axonal transport can be rescued by α-tubulin hyperacetylation (Dompierre et al., 2007). Loss or reduction in α-tubulin acetylation is associated with a number of neuropathological conditions, including familial dysautonomia, Alzheimer’s disease, Huntington’s disease, and Charcot-Marie-Tooth disease (Hempen and Brion, 1996; d'Ydewalle et al., 2001; Dompierre et al., 2007; Gardiner et al., 2007). Taken together, the injured axon requires numerous processes that are dependent on α-tubulin acetylation to initiate regrowth.

Previous studies have shown that α-tubulin K40 deacetylation is a primary and non-nuclear function of the class II zinc-dependent histone deacetylase (HDAC) family member, HDAC6 (Zhang et al., 2003; Zhang et al., 2008). By contrast, MEC-17/α-tubulin acetyltransferase-1 (αTAT1) is the enzyme responsible for α-tubulin K40 acetylation (Akella et al., 2010; Shida et al., 2010). Several studies to date have suggested important roles for HDAC6 and αTAT1 in regulating α-tubulin K40 acetylation and neurite outgrowth. In cultured neurons, pharmacological inhibition or knockdown of HDAC6 can prevent the inhibitory actions of MAG and CSPGs on axonal growth (Rivieccio et al., 2009). Similarly, it has been shown that αTAT1 is required for mechanosensation in *Caenorhabditis elegans* and that loss of αTAT1 leads to disruption of microtubule structural integrity and axonal morphologic defects in touch receptor neurons (Cueva et al., 2012; Topalidou et al., 2012). Moreover, the loss of αTAT1 disrupts axonal transport, leading to spontaneous axonal degeneration (Neumann and Hilliard, 2014). Studies in more complex organisms such as zebrafish and mice have shown that the loss of αTAT1 results in neuromuscular defects (Akella et al., 2010) and brain abnormalities, respectively (Kim et al., 2013).

Here, we demonstrate that αTAT1 plays an important role in the acetylation of α-tubulin required for axon growth. We show that in the presence of MAG or CSPGs, αTAT1 levels are reduced, resulting in decreased axonal α-tubulin K40 acetylation. This reduction in αTAT1 level is mediated via RhoA-ROCK signaling, is a result of decreased αTAT1 protein stability, and that reconstitution of αTAT1 by ROCK inhibition or lentiviral-mediated αTAT1 expression is sufficient to restore growth to MAG- and CSPGs-inhibited axons. In contrast to αTAT1, under these conditions HDAC6 levels and activity are unchanged following MAG and CSPGs exposure. Based on our data, we suggest a model of axon growth control through α-tubulin acetylation via the competing acetyltransferase and deacetylase activities of αTAT1 and HDAC6, respectively.

## Materials and Methods

### Antibodies and reagents

The following antibodies were used: CSPGs (2 μg/ml; CC117, EMD Millipore), cycloheximide (10 μg/ml; C0934, Sigma Aldrich), recombinant rat myelin-associated glycoprotein (MAG; 30 μg/ml; P07722, R&D Systems), Y-27632 ROCK inhibitor (10 μM; 1254, Tocris Bioscience), anti- αTAT1 (1:200; ab58742, Abcam), anti-HDAC6 (1:500; NB100-91805, Novus Biologicals), anti-acetylated α-tubulin (1:1000; D20G3, Cell Signaling Technology), anti-α-tubulin (1:5000; DM1A, Sigma-Aldrich), anti-β-actin (1:5000; AC-74, Sigma-Aldrich), anti-β III tubulin (1:5000; MRB_435_P, BioLegend) and anti-GFP (1:500; Sigma-Aldrich). Lentivirus containing GFP (control) or GFP-tagged wild-type *αTAT1* constructs, under the human cytomegalovirus (CMV) promoter, was purchased from Dr. Mingjie Li (Washington University School of Medicine, St. Louis, MO; Li et al., 2010). HDAC6 activity was determined using the fluorometric HDAC6 Activity Assay kit (BioVision), as per manufacturer’s instructions.

### Primary neurons

Fetuses of embryonic day 15.5 were obtained from timed pregnant female CD1 mice (Charles River). All animal procedures were performed in accordance with the Burke Medical Research Institute and Weill Cornell Medicine animal care committee’s regulations. Mouse primary neuronal cultures were obtained as described (Rivieccio et al., 2009). Briefly, neurons were allowed to adhere overnight before treatment at indicated concentration and duration (i.e., 30 min and 2 h). Lentiviral transduction conditions were optimized and were performed on neo-cortical cultures 2 d after plating (DIV 2) for 4 h of incubation, with no media change. Cultures were transduced with concentrated viruses at a multiplicity of infection of 5. Media were then replaced, and neurons were treated with CSPGs or MAG the next day for 24 h.

### Immunoblotting and immunocytochemistry

Protein lysates were prepared from cell cultures using RIPA buffer (Boston Bioproducts). Briefly, cells were grown in coated plates and rinsed with ice-cold PBS and centrifuged for 10 min at ≥16,000 × *g*. Pellet was collected and resuspended in RIPA buffer, and then further centrifuged for an additional 5 min at ≥16,000 × *g*. Protein concentration was determined by DC protein assay (5000112; Bio Rad). Immunoblot analysis was performed using a Li-Cor Odyssey system as described by Langley et al. (2008). For immunocytochemistry, primary cortical neurons were plated on poly-d-lysine (P6407; Sigma-Aldrich) wells and were fixed with 4% paraformaldehyde (BM-155-5; Boston BioProducts) for 10 min. Primary antibodies were used in conjunction with Alexa Fluor 488- or 594-conjugated secondary antibodies (1:2000; Invitrogen) for detection. Slides were mounted with ProLong anti-fade Gold reagent with DAPI (1:5000; Invitrogen). Immunostaining was examined under Carl Zeiss LSM 510 META confocal microscope for conventional single plane image. Image analyses were performed in Zen software (Carl Zeiss). All images were matched for exposure, gain, excitation power, and postprocessing. Localization analyses were performed using line scan profiling, and lines were drawn using ImageJ’s “line” tool that enable to measure peak intensity through the region of interest. To maintain consistency, neurite initiating segment (NIS) and distal region were measured 0.5 μm from the hillock and furthest end of the neurite (specified by Tuj1 positivity), respectively. The middle segment of the neurite was located to be half the length of the neurite. Intensities of acetylated α-tubulin and αTAT1 were normalized to total tubulin and Tuj1, respectively. For neurite length measurements, one longest neurite per neuron were measured from the cell body to end of the process labeled positively with Tuj1. For lentivirus overexpression experiments, only the neurites from GFP-positive neurons were measured.

### Real-time PCR

Total RNA preparation from cultured cells was performed as described in (Langley et al., 2008). TaqMan RNA-to-Ct one-step (4392938; Invitrogen) real-time PCRs were performed on total RNA as a duplex reaction using *αTAT1* gene expression assay (Mm00551286_m1; Applied Biosystems), and a *VIC*-labeled *β-actin* gene expression assay (4352341E; Applied Biosystems).

### Statistics

One- or two-way ANOVA, followed by the Bonferroni’s *post hoc* tests, or Student’s *t* tests were used to measure statistical significance; *p* < 0.05 was considered to be statistically significant.

## Results

### αTAT1 is downregulated by the axon growth inhibitory factors, CSPGs, and MAG

**Figure 1.**
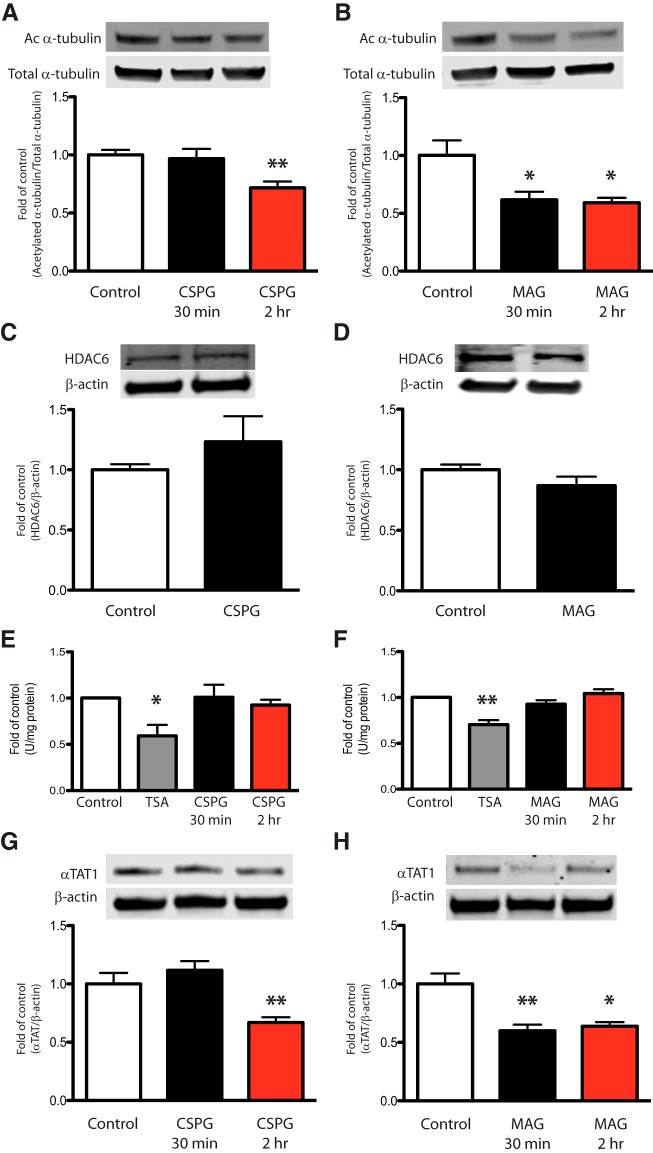
Growth inhibitory factors downregulate α-tubulin acetylation and αTAT1 levels. ***A***, ***B***, Immunoblot analysis of primary murine cortical neurons after exposure to soluble CSPGs (2 μg/ml; ***A***) or MAG (30 μg/ml; ***B***) showed a significant decrease in α-tubulin acetylation levels at the indicated times. Acetylated α-tubulin was normalized to total α-tubulin from the same immunoblot. ***C***, ***D***, Immunoblot analysis for HDAC6 after incubation with CSPGs (***C***) or MAG (***D***) for 2 h. HDAC6 level was normalized to β-actin from the same immunoblot. ***E***, ***F***, HDAC6 activity assays in primary neurons exposed to CSPGs (***E***) or MAG (***F***) after 30 min or 2 h did not change HDAC6 activity. Tubastatin A, a specific HDAC6 inhibitor, was used a positive control. ***G***, ***H***, Immunoblot analysis for αTAT1 after incubation with CSPGs (***G***) or MAG (***H***) for 30 min or 2 h showed a signification reduction in αTAT1 protein levels. αTAT1 level was normalized to β-actin from the same immunoblot. *, Significant downregulation compared to the control group *p* < 0.05; **p < 0.01 (one-way ANOVA followed by Bonferroni’s *post hoc* test was performed for ***A***, ***B***, ***E–H***. Student’s *t* test was performed for ***C***, ***D***).

CSPGs and MAG are well-characterized molecular barriers to axon regeneration following CNS injury. In the present study, we examined whether neuronal exposure to either CSPGs or MAG results in a change in α-tubulin acetylation levels. Cultured primary cortical neurons were treated with soluble CSPGs (2 μg/ml) or MAG (30 μg/ml) for 30 min or 2 h, harvested, and lysates assessed for α-tubulin acetylation by immunoblot analysis. Our results showed a significant decrease of α-tubulin acetylation within 30 min of exposure to MAG and within 2 h of exposure to CSPGs (Fig. 1*A*,*B*). Since α-tubulin acetylation level is determined by α-tubulin deacetylase and acetyltransferase activity, we examined HDAC6 and αTAT1 levels under these conditions. Immunoblot analysis for HDAC6 in lysates from CSPGs- or MAG-treated neurons showed no change in HDAC6 protein level (Fig. 1*C*,*D*). To determine whether HDAC6 activity, rather than level, contributed to the α-tubulin acetylation change by CSPGs and MAG, we examined HDAC6 enzymatic activity using fluorometric HDAC6 activity assay. No change in HDAC6 activity was observed in lysates from neurons exposed to either CSPGs or MAG (Fig. 1*E*,*F*). We then examined whether changes in α-tubulin acetylation were associated with changes in αTAT1 protein. Treatment with CSPGs or MAG significantly downregulated αTAT1 protein levels (Fig. 1*G*,*H*), and their effects were similar to the changes in α-tubulin acetylation with respect to time and magnitude (Fig. 1*A*,*B*). Taken together, these results indicate that the acute decrease in acetylation levels of α-tubulin in response to growth inhibitory factors is independent of HDAC6 levels and activity and can be attributed to a decrease in αTAT1 protein levels.

### Regulation of αTAT1 protein levels by CSPGs or MAG is ROCK dependent

**Figure 2.**
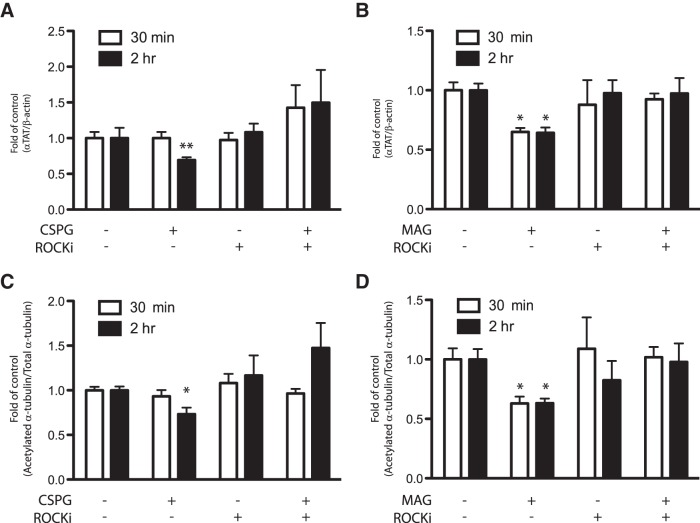
Downregulation of αTAT1 and α-tubulin acetylation by CSPGs and MAG is mediated through ROCK-dependent pathway. Primary cortical neurons were treated with either CSPGs (2 μg/ml) or MAG (30 μg/ml) at indicated times, with or without ROCK inhibitor (Y-27632; 10 μM). ***A***, ***B***, Immunoblot analysis for αTAT1 showed that ROCK inhibitor prevented downregulation of αTAT1 after exposure to CSPGs (***A***) and MAG (***B***). αTAT1 level was normalized to β-actin from the same immunoblot. ***C***, ***D***, Immunoblot analysis for acetylated α-tubulin showed that ROCK inhibitor also prevented CSPGs- and MAG-induced (***C***, ***D***, respectively) reduction of α-tubulin acetylation. Acetylated α-tubulin was normalized to total α-tubulin from the same immunoblot. *, Significant downregulation compared to the control group at their respective times, *p* < 0.05; **p < 0.01 (two-way ANOVA followed by Bonferroni’s *post hoc* test was performed).

It is well established that MAG and CSPGs exert growth inhibitory effects via distinct receptors. For instance, MAG has been shown to activate the small GTPase RhoA via NogoR (Fournier et al., 2001; Domeniconi et al., 2002; Liu et al., 2002; Wang et al., 2002b) and p75 neurotrophin (Wang et al., 2002a; Wong et al., 2002; Yamashita et al., 2002) receptors, leading to subsequent activation of RhoA and its downstream kinase, ROCK (Dergham et al., 2002; Yamashita et al., 2002; Fournier et al., 2003). Although CSPGs use PTPσ to activate yet unidentified pathways (Shen et al., 2009), studies have shown that the RhoA/ROCK pathway also mediates the neurite growth-inhibitory activity of CSPGs (Dergham et al., 2002; Borisoff et al., 2003; Monnier et al., 2003). Since the inhibitory signals of CSPGs and MAG may converge on the RhoA/ROCK pathway, we next delineated the mechanism of action whereby CSPGs or MAG regulates αTAT1. Primary cortical neurons were cotreated with CSPGs or MAG, and Y-27632, a well-established ROCK inhibitor. Consistent with our prior observations (Fig. 1*A*,*B*), CSPGs and MAG reduced αTAT1 protein levels (Fig. 2*A*,*B*). Cotreatment with the ROCK inhibitor prevented this effect (Fig. 2*A*,*B*). Furthermore, the reduction in α-tubulin acetylation was prevented when both the ROCK inhibitor and either growth inhibitory substrates were applied (Fig. 2*C*,*D*). In line with our observations in Figure 1*C*,*D*, no changes in HDAC6 protein levels were seen under these conditions (data not shown). These findings indicate that αTAT1 regulation by CSPGs and MAG is ROCK dependent.

### αTAT1 downregulation by CSPGs and MAG predominantly occurs in the middle and distal regions of neurites

**Figure 3.**
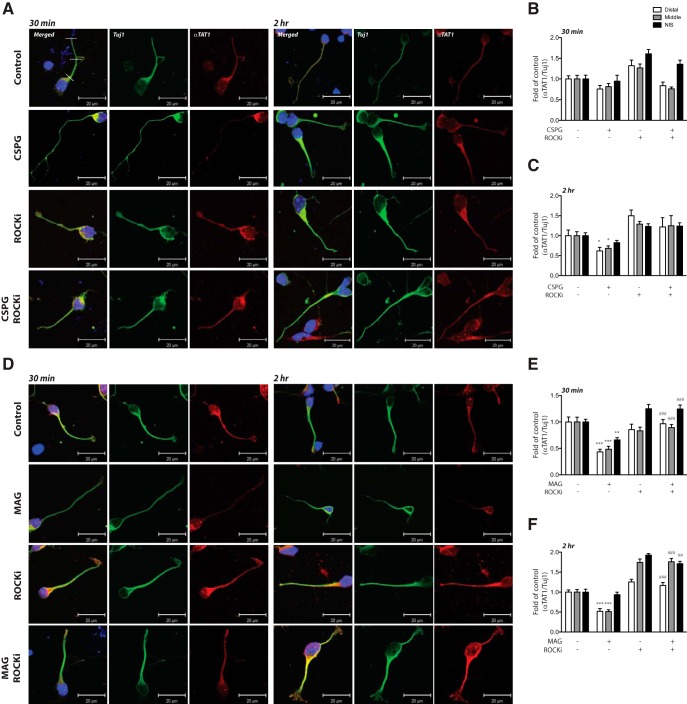
CSPGs and MAG change neurite αTAT1 expression. ***A***, ***D***, Confocal immunofluorescent micrographs showing the distribution of αTAT1 in cortical neurons after exposure to growth inhibitory factors CSPGs (2 μg/ml; ***A***) or MAG (30 μg/ml; ***D***) with or without ROCK inhibitor (Y-27632; 10 μM) after 30 min and 2 h. Immunolabeling was performed using antibodies against αTAT1 (1:200; red) and Tuj1 (1:5000; green). Nuclei of neurons were labeled with DAPI (blue). Immunofluorescence intensity at different regions of the axon as indicated by white dashed line (i.e., distal, middle, and NIS) was quantified in ***B***, ***C*** and ***E***, ***F***. *, Treatment versus control *p* < 0.05; **, treatment versus control *p* < 0.01; ***, treatment versus control *p* < 0.001; ##, cotreatment with MAG and ROCKi versus MAG alone *p* < 0.01; ###, cotreatment with MAG and ROCKi versus MAG alone *p* < 0.001 (two-way ANOVA followed by Bonferroni’s *post hoc* test was performed). Scale bar, 20 μm.

**Figure 4.**
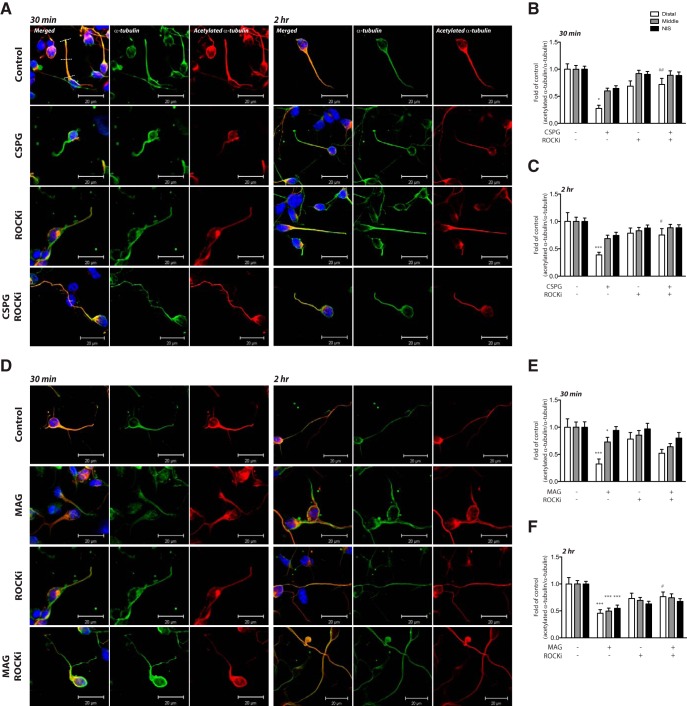
CSPGs and MAG change neurite α-tubulin acetylation. ***A***, ***D***, Confocal immunofluorescent micrographs showing the distribution of αTAT1 in cortical neurons after exposure to growth inhibitory factors CSPGs (2 μg/ml; ***A***) or MAG (30 μg/ml; ***D***) with or without ROCK inhibitor (Y-27632; 10 μM) after 30 min and 2 h. Immunolabeling was performed using antibodies against acetylated α-tubulin (1:1000; red) and α-tubulin (1:5000; green). Nuclei of neurons were labeled with DAPI (blue). Immunofluorescence intensity at different regions of the axon as indicated by white dashed line (i.e., distal, middle, and NIS) was quantified in ***B***, ***C*** and ***E***, ***F***. *, Treatment versus control *p* < 0.05; ***, treatment versus control *p* < 0.001; #, cotreatment with ROCKi versus treatment alone *p* < 0.05; ##, cotreatment with ROCKi versus treatment alone *p* < 0.01 (two-way ANOVA followed by Bonferroni’s *post hoc* test was performed). Scale bar, 20 μm.

In addition to measuring global changes of αTAT1 levels in cortical neurons via immunoblotting, we further examined the effects of CSPGs and MAG on αTAT1 levels in different regions of neurites. Primary cortical neurons were cultured for 24 h, treated with soluble CSPGs or MAG for 30 min or 2 h, and immunostained for αTAT1. Our immunostaining results indicated that in control neurons, αTAT1 was evenly distributed from the cell body to the distal end of the neurite. Consistent with previous studies (Shida et al., 2010), αTAT1 was not localized to the nucleus of cortical neurons. By contrast, a 2-h exposure to CSPGs resulted in a significant reduction in αTAT1 intensity in the middle and distal regions of neurites (Fig. 3*A–C*). Similarly, exposure to MAG resulted in significant reduction in the middle and distal regions of the neurite; however, this reduction occurred within 30 min and was also seen in the proximal (NIS) region of the neurite (Fig. 3*D–F*). Administration of the ROCK inhibitor, Y-27632, alone did not significantly alter αTAT1 localization compared with control neurons, but when co-administered with CSPGs or MAG it prevented the αTAT1 reduction in the neurites. Immunostaining using an antibody against acetylated α-tubulin revealed a similar pattern of α-tubulin acetylation change to that observed for αTAT1. Significant decreases in acetylated α-tubulin were predominantly seen in distal to middle regions with CSPGs (Fig. 4*A–C*) or MAG (Fig. 4*D–F*) treatment. The distal neurite α-tubulin acetylation decrease by CSPGs was attenuated by cotreatment with the ROCK inhibitor at 30 min and 2 h, while the decrease by MAG was attenuated by cotreatment with the ROCK inhibitor at 2 h (Fig. 4). Attenuation of α-tubulin acetylation decrease by MAG at 30 min did not reach a level of significance (Fig. 4*E*).

### αTAT1 downregulation by CSPGs and MAG correlates with decreased neurite length

**Figure 5.**
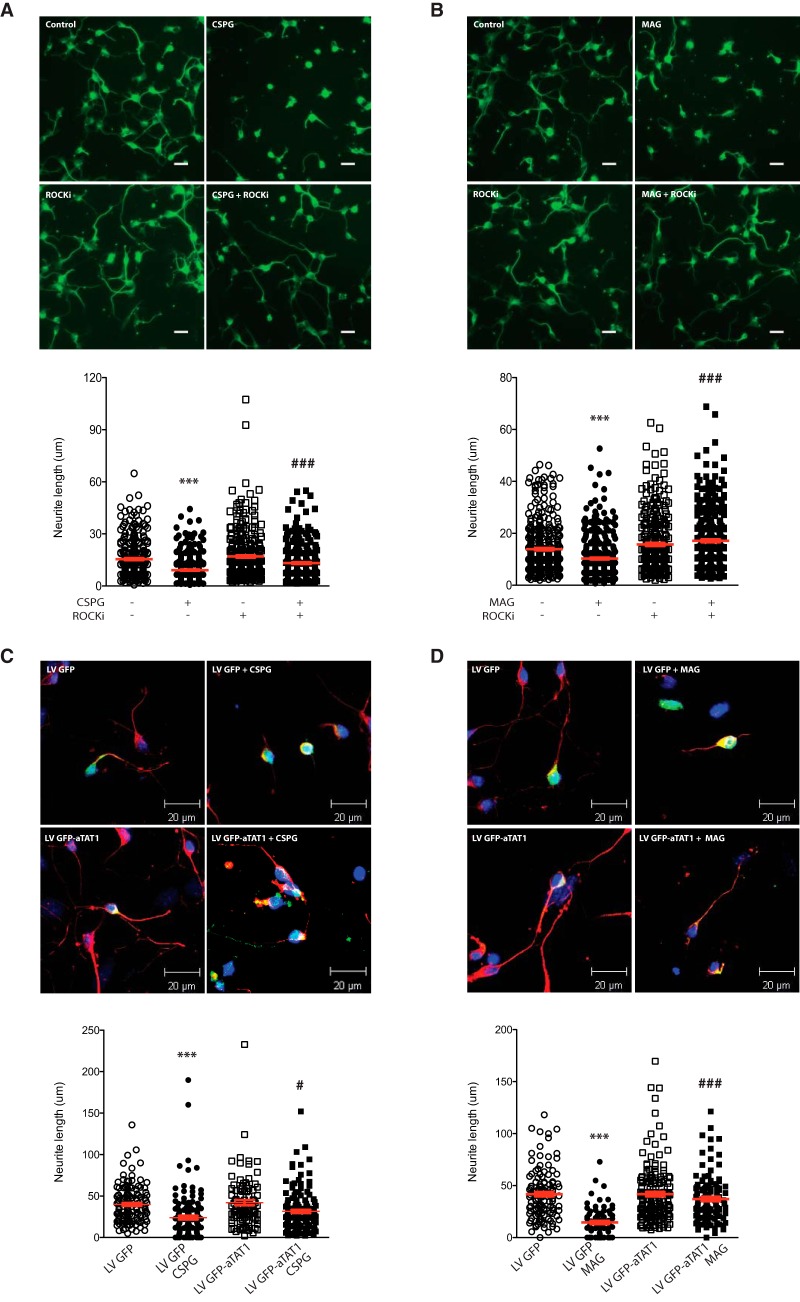
ROCK inhibition and overexpression of αTAT1 reverse CSPGs- and MAG-induced inhibition of neurite outgrowth. ***A***, ***B***, Fluorescent microscopy of primary cortical neurons incubated with CSPGs (2 μg/ml; ***A***) or MAG (30 μg/ml; ***B***), with or without ROCK inhibitor (Y-27632; 10 μM) for 24 h. Neurite lengths and mean neurite length for each condition are shown in column scatter plots below micrographs. ***, CSPGs or MAG treatment versus untreated control *p* < 0.001; ###, cotreatment with ROCKi versus treatment alone *p* < 0.001 (one-way ANOVA followed by Bonferroni’s *post hoc* test was performed). Scale bar, 10 μm (***A***, ***B***). ***C***, ***D***, Confocal immunofluorescent microscopy of primary cortical neurons following infection with lentiviral GFP (LV GFP; control) or lentiviral GFP-*αTAT1* (LV GFP-*αTAT1*) with or without CSPGs (2 μg/ml; ***C***) or MAG (30 μg/ml; ***D***). Transduced neurites were identified by immunolabeling with antibodies for neuron-specific Tuj1 (1:5000; red) and GFP (1:500; green) and quantified with ImageJ software. Neurite lengths and mean neurite length for each condition are shown in column scatter plots below micrographs. ***, CSPGs or MAG treatment versus untreated control *p* < 0.001; # and ###, LV GFP-*αTAT1* with CSPGs or MAG versus LV GFP with CSPGs or MAG, *p* < 0.05 and *p* < 0.001, respectively (two-way ANOVA followed by Bonferroni’s *post hoc* test was performed). Scale bar, 20 μm (***C***, ***D***).

Based on our observations that CSPGs and MAG decrease αTAT1 expression and α-tubulin acetylation, we hypothesized that αTAT1 decrease is responsible for the inhibition of neurite outgrowth. To test this hypothesis, we examined the effects of CSPGs and MAG on neurite length in the presence or absence of ROCK inhibitor. Cultured primary cortical neurons were plated, cultured for 4 h, then treated with soluble CSPGs (2 μg/ml) or MAG (30 μg/ml) in the presence of the ROCK inhibitor, Y-27632, for 24 h. Consistent with our previous findings (Rivieccio et al., 2009), and our current findings that CSPGs and MAG decrease αTAT1 and α-tubulin acetylation levels, treatment with either CSPGs (Fig. 5*A*) or MAG (Fig. 5*B*) significantly reduced neurite length in cortical neurons (42% and 25% reduction, respectively). Cotreatment with ROCKi restored neurite growth (44% compared to CSPGs treatment alone; 66% compared to MAG treatment alone) indicating that the axon growth inhibitory effect of either CSPGs (Fig. 5*A*) or MAG (Fig. 5*B*) was dependent on ROCK. To demonstrate a causative relationship for reduced αTAT1 and inhibited neurite growth, we reconstituted αTAT1 expression to CSPGs- or MAG-treated neurites. Primary cortical neurons (DIV 2) were infected with *αTAT1*-GFP-lentivirus or GFP-lentivirus (control) for 4 h. Media were then replaced, and neurons were treated with CSPGs or MAG for 24 h. Assessments of neurite length from infected (GFP-positive) cortical neurons show that *αTAT1*-lentivirus-mediated overexpression of αTAT1 significantly reversed the growth inhibitory effects of CSPGs and MAG (Fig. 5*C*,*D*, respectively; 80% compared CSPGs treatment alone, and 169% relative to MAG treatment alone).

### CSPGs- and MAG-induced αTAT1 decrease occurs via a change in αTAT1 protein stability

**Figure 6.**
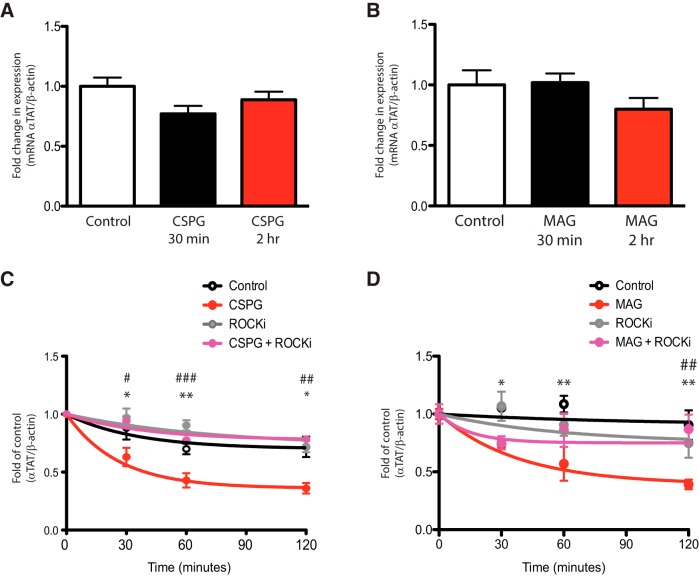
αTAT1 protein stability is reduced in cortical neurons treated with CSPGs or MAG. ***A***, ***B***, Bar graphs showing real-time quantitative RT-PCR results from primary cortical neurons incubated with CSPGs (2 μg/ml; ***A***) or MAG (30 μg/ml; ***B***) for 30 min or 2 h reveal no change in *αTAT1* mRNA. ***C***, ***D***, Cycloheximide chase assay graphs showing αTAT1 protein degradation in primary cortical neurons over time after protein translation inhibition with cycloheximide (10 μg/ml). Neurons were treated with or without CSPGs (2 μg/ml; ***C***) or MAG (30 μg/ml; ***D***) and cotreated with or without ROCK inhibitor (Y-27632; 10 μM). *, Treatment with growth inhibitory substrate versus 0 min *p* < 0.05; **, treatment with growth inhibitory substrate versus 0 min, *p* < 0.01; #, CSPGs or MAG cotreatment with ROCKi versus CSPGs or MAG treatment alone at 30 min, *p* < 0.05; ###, CSPGs or MAG cotreatment with ROCKi versus CSPGs or MAG treatment alone at 60 min, *p* < 0.001; ##, CSPGs or MAG treatment with ROCKi versus CSPGs or MAG treatment alone at 120 min, *p* < 0.01 (two-way ANOVA followed by Bonferroni’s *post hoc* test was performed).

The observed downregulation of αTAT1 in neurites treated with CSPGs or MAG could occur via changes in *αTAT1* transcription or αTAT1 protein stability. To determine whether transcription of *αTAT1* is decreased with CSPGs or MAG treatment, primary cortical neurons were treated with CSPGs or MAG for 30 min or 2 h, harvested and analyzed for *αTAT1* expression by quantitative RT-PCR. No significant changes in *αTAT1* mRNA levels were observed in any of the conditions (Fig. 6*A*,*B*), suggesting that the reduction in αTAT1 protein levels in response to growth inhibitory factors is dependent on *αTAT1* transcription. To determine whether the changes αTAT1 reflect in a change in protein stability, we performed cycloheximide chase assays in CSPGs or MAG-treated primary neurons. In cycloheximide-treated (10 μg/ml) control neurons, the levels of αTAT1 protein remained relatively steady over the 2-h course of the experiment (Fig. 6*C*,*D*). By contrast, we saw a significant reduction in αTAT1 protein levels within 30 min with CSPGs (Fig. 6*C*) or MAG (Fig. 6*D*), which persisted at the 2-h time point. Similar to controls, the cotreatment of neurons with cycloheximide and the ROCK inhibitor, Y-27632, resulted in no significant changes in αTAT1 levels during the 2-h course of the experiment; however, cotreatment with Y-27632 could prevent αTAT1 protein decrease observed by CSPGs (Fig. 6*C*) or MAG treatment alone (Fig. 6*D*). These observations strongly suggest that the reduction in αTAT1 seen with MAG or CSPGs treatment is due to a ROCK-dependent increase in the turnover rate of this protein.

## Discussion

Previous studies have established that CSPGs and MAG play critical roles in the extrinsic inhibition of axon regeneration following CNS injury. Thus, they have been widely studied, both *in vitro* and *in vivo*, to identify of molecular targets that can be manipulated to overcome CNS regeneration failure, with the ultimate goal of reducing dysfunction and disability. Previous studies have highlighted the role of HDAC6 in mediating the growth inhibitory effects of MAG and CSPGs. Moreover, these studies identified HDAC6 as a novel target for pharmacological inhibition or genetic downregulation using small molecule inhibitors or siRNAs, respectively, which can promote neurite outgrowth in multiple models of growth inhibition using MAG and CSPGs (Rivieccio et al., 2009).

In this study, we show that the microtubule protein, α-tubulin, which is one of the most recognized intracellular protein targets of HDAC6, is deacetylated in neurons following stimulation by CSPGs or MAG (Fig. 1*A*,*B*). This deacetylation was most striking in the distal portion of neurites, but also occurred in the middle and proximal regions (Fig. 3*A–F*). Given that HDAC6 inhibition can rescue neurite outgrowth in CSPGs- or MAG-stimulated neurons and can increase α-tubulin acetylation (Rivieccio et al., 2009), we thought it logical that CSPGs or MAG might regulate α-tubulin acetylation via HDAC6; however, under these conditions, we saw no evidence for altered HDAC6 levels or its enzymatic activity downstream of CSPGs or MAG signaling (Fig. 1*C–F*). By contrast, under the same growth inhibitory conditions, our findings reveal that αTAT1 levels were significantly downregulated (Fig. 1*G*,*H*). Since α-tubulin acetylation is regulated by the opposing activities of HDAC6 (deacetylase) and αTAT1 (acetyltransferase), our results suggest that αTAT1 regulation is the main driver of CSPGs- or MAG-induced α-tubulin acetylation loss in the neurite. This notion is highly supported by our findings that αTAT1 downregulation is both temporally and spatially identical to α-tubulin acetylation changes downstream of MAG or CSPGs treatment (Figs. 3, 4), and that αTAT1 reconstitution by lentiviral-αTAT1 infection can overcome neurite growth inhibition (Fig. 5*C*,*D*). These findings are also supported by the recent demonstration that overexpression of αTAT1 in DRG neurons significantly increases α-tubulin acetylation toward the distal portion of the axon and significantly increases axon length (Lin et al., 2017). Furthermore, that α-tubulin acetylation level is dependent on αTAT1 is consistent with a recent report demonstrating that αTAT1 is highly expressed in mouse brain tissue, and that αTAT1 deletion results in a near absence of acetylated α-tubulin (Kim et al., 2013).

Our study, herein, also gives insight into how αTAT1 is regulated downstream of MAG and CSPGs signaling as a reduction in αTAT1, and consequently α-tubulin acetylation, can be prevented by inhibiting the RhoA-ROCK pathway (Figs. 2–4). Several studies have identified that MAG and CSPGs exert their axon growth inhibitory effects via a receptor complex comprising Nogo receptor family members and p75NTR low-affinity neurotrophin receptors that in turn signal via the receptor-bound GTPase, RhoA. A well-characterized canonical downstream effector of RhoA is Rho-associated protein kinase, ROCK, which is involved in many aspects of neuronal functions including neurite outgrowth and retraction. As such, the axon growth-inhibitory effects of MAG and CSPGs can be reversed by blockade of the Rho-ROCK pathway *in vitro* and *in vivo* (Borisoff et al., 2003; Mimura et al., 2006; Hur et al., 2011).

The relationship between ROCK and acetylation of α-tubulin has been underscored by studies in mice overexpressing αTAT1 that is deficient of catalytic activity but not α-tubulin binding. These mutant animals have less acetylated α-tubulin and enhanced microtubule depolymerization sensitivity to nocodazole (Kalebic et al., 2013a,b), a well-established activator of RhoA-ROCK pathway (Krendel et al., 2002; Chang et al., 2008). Here, we also reveal that activation of the RhoA-ROCK pathway by CSPGs and MAG act to decrease αTAT1 levels by decreasing its stability at a posttranslational level (Fig. 6). How RhoA-ROCK pathway regulates αTAT1 protein levels is still an open question. Previous studies established that RhoA has protein degradation effects, in particular, on p27^kip^ through regulation of cyclin E/CDK2 activity (Hirai et al., 1997; Hu et al., 1999). Expression of dominant-negative RhoA inhibited p27^kip^ degradation *in vitro* (Hu et al., 1999). In addition to the traditional roles of RhoA on actin dynamics, our study and others strongly suggests that RhoA has a role in regulating protein degradation. While the mechanism behind RhoA signaling and αTAT1 degradation remains elusive, this finding nevertheless places emphasis on the importance of events downstream of the Rho-ROCK pathway and identifying the stabilization/destabilization domain(s) of αTAT1 for drug development targets in promoting CNS axon regeneration in the presence of growth impeding factors such as CSPGs and MAG.

A critical question that remains is whether or not α-tubulin acetylation is required for neurite growth. In neurons, microtubule arrays are constantly adapted to fit their physiologic needs by modulating the balance between dynamic short-lived, and stable long-lived microtubule sub-populations. During neurite extension, the more stable microtubules are needed in the proximal axon to drive forward growth, but those in the axon tip/growth cone must be highly dynamic if it is to grow and respond to extracellular stimuli (Tahirovic and Bradke, 2009; Bradke et al., 2012). Microtubule structure, organization, stability, and function are highly regulated by microtubule-associated proteins (MAPs) and posttranslational modifications. Spatial localizations of acetylated α-tubulin along axons reflects differences in their stability, with enriched acetylation in the more long-lived or stabilized microtubule populations that predominate in the proximal axon region and low-level acetylation at neurite tips (Black et al., 1989; Webster and Borisy, 1989; Brown et al., 1992; Baas et al., 1993). It may be that stabilizing microtubules enables the tip of the axon push through what would be negative growth signals in the injured nervous system. This hypothesis would be consistent with recent findings, where taxol has been shown to stabilize microtubules and augment regeneration of injured optic nerve (Sengottuvel et al., 2011; Sengottuvel and Fischer, 2011) and injured spinal cord axons (Hellal et al., 2011).

In addition to microtubules serving as architectural elements that shape the elongation of growing axons, and they are key components of the machinery that transports mitochondria and material required for axon growth from their sites of synthesis in the cell body into the axon (Yogev et al., 2016). Several studies have revealed that microtubule acetylation affects the affinity and progressivity of microtubule motors, playing a positive role in motor-based trafficking in axons (Reed et al., 2006; Dompierre et al., 2007; Hammond et al., 2010; Alper et al., 2014; Godena et al., 2014). Dompierre et al., proposed that the neurodegenerative Huntington disorder might involve a defect in tubulin acetylation, and that increasing tubulin acetylation can enhance the recruitment of the molecular motors dynein and kinesin-1 to microtubules to promote vesicular transport in differentiated neurons (Dompierre et al., 2007). Thus, the role of α-tubulin acetylation by αTAT1 in neurite extension might be to facilitate growth-requiring cargo delivery.

It is important to consider that we cannot exclude the possibility that the roles of αTAT1 and HDAC6 in axon regeneration are independent of α-tubulin and/or their acetyltransferase and deacetylase activities, respectively. A recent study by Lin et al. (2017) found that while αTAT1 overexpression in DRGs increases axonal α-tubulin acetylation in cultured DRG neurons, the overexpression of a catalytically inactive mutant, αTAT1-D157N, does not. Yet both the catalytically active and inactive αTAT1s significantly increased axonal lengths *in vitro*. Similarly, with regard to α-tubulin acetylation and microtubule stability, findings by Kalebic et al. (2013b) revealed that it is the interaction of αTAT1 with microtubules, and not acetylation per se, that is the critical factor regulating microtubule stability. Nevertheless, our findings here demonstrate an exciting and novel role for αTAT1 as a critical acute mediator of axon growth that is regulated downstream of CSPGs and MAG, and the RhoA/ROCK signaling cascade, which is a known molecular target to promote axon regeneration. In addition to this previously unidentified role, our work suggests that protecting αTAT1 stability/levels may provide an additional robust strategy to overcome axonal regeneration failure after CNS injury. Furthermore, the interplay between αTAT1 and HDAC6 in the context of α-tubulin acetylation will be an interesting area of future exploration. One can surmise that when αTAT1 is downregulated at the axonal tips, HDAC6 may become the predominant enzyme and promotes α-tubulin deacetylation. Studies are under way to explore the spatio-temporal relationship between these two opposing enzymes in regulating axonal growth, as are studies to define the role of αTAT1 *in vivo*, especially in animal models of traumatic brain injury and spinal cord injury.

## Synthesis

Reviewing Editor: Quentin Pittman, University of Calgary Decisions are customarily a result of the Reviewing Editor and the peer reviewers coming together and discussing their recommendations until a consensus is reached. When revisions are invited, a fact-based synthesis statement explaining their decision and outlining what is needed to prepare a revision will be listed below. The following reviewer(s) agreed to reveal their identity: Larry Benowitz, Roman Giger

Overall, the reviewers believe that have succeeded in addressing the commnets and the manuscript is considerably stronger. However, there are several more things are needed before the paper can be accepted for publication. These are largely requests for clarification and for modification of your conclusions to better reflect the data. Please carefully consider these and modify the manuscript appropriately. There is no need for further experiments and I will be dealing with your revision ( and any rebuttals) personally. The comments follow:

The authors overstate their case in several places. For example,

1. In Fig. 4E, the effect of the ROCK inhibitor was not significant (MAG/ROCKi, 30 min). The authors should state this clearly rather than bundle all of the data into a statement of a general trend. In Fig. 4F, the ROCK inhibitor alone seems to have decreased levels of acetylated a-tubulin (third set of bars). Any thoughts about this?

2. In Fig. 5B (graph), the effect of the ROCK inhibitor in restoring outgrowth in the presence of CSPGs seems small, though the symbols indicate a high level of statistical significance. The authors should mention the percent increase in outgrowth in the text.

3. Similarly, in the graph of Fig. 5C, the magnitude of the increase in neurite growth seems small despite attaining statistical significance. The actual percentage increase should be stated in the text.

4. Fig. 6B, although the decrease in aTAT1 mRNA is not reported to be significant, it does appear to decline by ~20%, and a change of that magnitude in mRNA could conceivably be responsible for a 50% decrease in levels of the protein.

5. At the end of the Discussion, the authors address the clinical importance of their results, “In addition to this previously unidentified role, our work suggests that protecting αTAT1 stability/levels may provide an additional robust strategy to overcome axonal regeneration failure after CNS injury.” There are already well established inhibitors of the RhoA-ROCK pathway, so why would protecting the stability of aTAT1 be superior?

Other points:

1. Why do the investigators use soluble MAG and CSPG instead of substrate-bound proteins, which is more physiologically relevant and is commonly done? Do neurons respond similarly to MAG and CSPG regardless of whether they are substrate bound?

2. P. 10, the authors state “In agreement with our prior observations, no changes in HDAC6 protein levels were seen under these conditions (data not shown).” What prior observations? A reference should be cited.

3. The paper's authors are concealed, but the animal statement and a reference to their prior work suggests that they are at Burke/Cornell, where another group recently reported that the effects of cell-extrinsic inhibitors to growth are mediated largely through PARP1: “exposure of neurons to growth-limiting molecules--such as myelin-derived Nogo and myelin-associated glycoprotein--or reactive astrocyte-produced chondroitin sulfate proteoglycans activates PARP1, resulting in the accumulation of poly(ADP-ribose) in the cell body and axon and limited axonal growth. Accordingly, we find that pharmacological inhibition or genetic loss of PARP1 markedly facilitates axon regeneration over nonpermissive substrates.” Oddly, that paper is not cited here, leaving the reader wondering what the connection might be between the two datasets.
